# Supplementary material for: Genetic meta-analysis of levodopa induced dyskinesia in Parkinson’s disease
Source: NPJ Parkinsons Dis. 2023 Aug 31;9:128. doi: 10.1038/s41531-023-00573-2 (PMC10471743; doi:10.1038/s41531-023-00573-2)
Supplement: Supplementary file 1 — Supplementary Material [file 41531_2023_573_MOESM1_ESM.pdf]

Supplementary Online Content

1. eTables

eTable 1. Study sample sizes and genotyping array.

eTable 2. List of covariates added on both the basic and adjusted model across cohorts

eTable 3. Genomic inflation values in each cohort and meta-analysis summary statistics

eTable 4. Sensitivity analyses lead SNP P-values in the basic CPH model for the TPD cohort

eTable 5. Lead SNP P-values in the CPH model including and excluding PDBP cohort in the basic and adjusted models

eTable 6. List of fine-mapped consensus SNPs on each locus.

eTable 7. Colocalization hypotheses posterior probabilities

eTable 8. Candidate variants analysis.

eTable9. List of DJ-1, and PARKIN intergenic variants with a significance value lower than 0.05 on the raw GWAS meta-analysis

eTable 10. Table of SNPs used to derive the GRS in the TPD cohort

eTable 11. MoCa and UPDRS score comparison between PD-LiD and PD groups

2. eFigures

eFigure 1. Quality control flowchart.

eFigure 2. LiD risk factors Kaplan Meyer curves.

eFigure 3. Power calculation and simulation.

eFigure 4. SNP metrics correlation between the CPH basic and best models.

eFigure 5. LRP8 locus fine-mapping and top 5 TFBS marks.

eFigure 6. XYLT1 locus fine-mapping and brain cell type specific regulatory marks.

eFigure 7. XYLT1 locus fine-mapping and top 5 TFBS marks.

eFigure 8. PRS Receiver operating characteristic (ROC) curves.

eFigure 9. Precision-recall curves (PROC) and the area under the curve for unweighted data.

3. eData

eData 1. List of all covariates screened in the stepwise regression model

1. eTables.

eTable 1. Study sample sizes and genotyping array.

Study Name	Samples source	Abbreviations	N	Genotyping array
Tracking Parkinson's Disease	UK	TPD	2000	Illumina HumanCoreExome array
Oxford Parkinson's Disease Centre Discovery Cohort	UK	OPDC	1082	Illumina HumanCoreExome-12 v1.1 or Illumina Infinium HumanCoreExome-24 v1.1
Parkinson's Progression Markers Initiative	USA	PPMI	415	WGS
Advancing Parkinson's Disease Biomarkers Discovery	USA	PDBP	873	WGS
Simvastatin as a neuroprotective treatment for Parkinson's disease	UK	PD-STAT	174	Illumina Neurochip

WGS = Whole Genome Sequencing

eTable 2. List of covariates added on both the basic and adjusted model across cohorts

Study Name	Covariates in basic model	Covariates in the adjusted model
Tracking Parkinson’s Disease	AAO, SEX, 5 PCs	AAO, SEX, 5 PCs, BASELINE DISEASE DURATION, BASELINE MDS-UPDRS-III total, BASELINE L-DOPA DOSE
Oxford Parkinson's Disease Centre Discovery Cohort	AAO, SEX, 5 PCs	AAO, SEX, 5 PCs, BASELINE MDS-UPDRS-III total, BASELINE LEDD
Parkinson's Progression Markers Initiative	AAO, SEX, 5 PCs	AAO, SEX, 5 PCs, BASELINE MDS-UPDRS-III total, BASELINE DISEASE DURATION
Advancing Parkinson’s Disease Biomarkers Discovery	AAD, SEX, 5 PCs	AAD, SEX, 5 PCs, BASELINE MDS-UPDRS III total, BASELINE DISEASE DURATION
Simvastatin as a neuroprotective treatment for Parkinson’s disease	AAO, SEX, 5 PCs	AAO, SEX, 5 PCs

eTable 3. Genomic inflation values in each cohort and meta-analysis summary statistics.

Cohort Name	Lambda
TPD	1.02
OPDC	0.99
PPMI	0.97
PDBP	0.87
PD-STAT	0.73

eTable 4. Sensitivity analyses lead SNP P-values in the basic CPH model for the TPD cohort

CHR	BP	SNP	A1	A2	Basic model P-value	Levodopa model P-value	MDS-UPDRS III model P-value
1	53778300	rs72673189	A	G	1.96E-04	2.39E-04	2.50E-04
4	32435284	rs189093213	A	G	6.32E-03	1.89E-03	3.49E-03
16	17044975	rs180924818	G	A	1.69E-04	2.62E-04	1.21E-04

Basic model P-value = P-value on the basic model without confounding variables
Levodopa model P-value = Levodopa dose at the time point the outcome was met
MDS-UPDRS III model P-value = MDS-UPDRS III total score at the time point the outcome was met.

eTable 5. Lead SNP P-values in the CPH model including and excluding PDBP cohort in the basic and adjusted models

CHR:POS	SNP	A1	A2	MAF	HR	SE	P-value	N	PDBP INCLUDED	MODEL
4:32435284	rs189093213	A	G	0.02	3.08	0.19	1.673e-09	2687	YES	ADJUSTED
4:32435284	rs189093213	A	G	0.02	2.73	0.18	6.154e-08	2784	YES	BASIC
4:32435284	rs189093213	A	G	0.02	3.29	0.19	6.24e-10	2446	NO	ADJUSTED
4:32435284	rs189093213	A	G	0.02	2.88	0.19	2.989e-08	2543	NO	BASIC
16:17044975	rs180924818	A	G	0.98	0.32	0.20	6.265e-09	2687	YES	ADJUSTED
16:17044975	rs180924818	A	G	0.98	0.35	0.19	8.197e-08	2784	YES	BASIC
16:17044975	rs180924818	A	G	0.98	0.32	0.20	6.265e-09	2446	NO	ADJUSTED
16:17044975	rs180924818	A	G	0.98	0.35	0.19	8.197e-08	2543	NO	BASIC
1:53778300	rs72673189	A	G	0.0	2.76	0.18	1.527e-08	2610	YES	ADJUSTED
1:53778300	rs72673189	A	G	0.02	2.72	0.18	2.654e-08	2707	YES	BASIC
1:53778300	rs72673189	A	G	0.02	2.93	0.19	1.505e-08	2369	NO	ADJUSTED
1:53778300	rs72673189	A	G	0.02	2.83	0.19	4.214e-08	2466	NO	BASIC

eTable 6. List of fine-mapped consensus SNPs on each locus.

SNP	CHR	P-value	Effect	SE	A1	A2	MAF	leadSNP	ABF.PP	FINEMAP.PP	SUSIE.PP	POLYFUN_SUSIE.PP	Support	mean.PP	mean.CS	Locus
rs72673189	1	1.5e-08	1.01	0.18	A	G	0.02	TRUE	1	0	1	0	2	0.50	0	LRP8
rs180924818	16	6.2e-09	-1.14	0.20	A	G	0.02	TRUE	1	1	1	1	4	1	1	XYLT1
rs137895239	16	3.1e-05	0.88	0.21	A	G	0.02	FALSE	0	1	1	1	3	0.75	0	XYLT1
rs142441980	16	1.4e-06	0.88	0.18	A	G	0.03	FALSE	0.01	1	1	1	3	0.75	0	XYLT1
rs17207399	16	2e-04	-0.47	0.13	C	G	0.07	FALSE	1	1	1	1	3	0.75	0	XYLT1
rs189093213	4	1.7e-09	1.12	0.19	A	G	0.01	TRUE	0.61	0.96	1	1	3	0.90	0	LINC02353
rs10023843	4	0.1	0.55	0.36	T	G	0.02	FALSE	0	0	1	1	2	0.50	0	LINC02353
rs139511855	4	4.5e-05	-1.09	0.27	A	G	0.01	FALSE	0	0	1	1	2	0.50	0	LINC02353
rs147573196	4	2.5e-06	1.20	0.25	A	T	0.01	FALSE	0	0	1	1	2	0.50	0	LINC02353
rs28858724	4	0.03	-0.34	0.17	A	G	0.04	FALSE	0	0	1	1	2	0.50	0	LINC02353

leadSNP: Whether a given SNP is the locus lead SNP.
<tool>.CS: The posterior probability that a SNP is casual of the LiD phenotype.
Support: The number of fine-mapping tools that nominated the Consensus SNP
mean.PP: The mean SNP wise PP across fine mapping tools
mean.CS: If mean PP is greater than the 95% probability threshold (mean.PP > 0.95), then mean.CS is 1, else 0

eTable 7.Colocalization hypotheses posterior probabilities

ENSEMBL ID	HGNC	nsnps	PP.H0	PP.H1	PP.H2	PP.H3	PP.H4	ratio_PPH4_PPH3
ENSG00000162616	DNAJB4	4840	6.76E-05	7.03E-05	0.23	0.24	0.52	2.17
ENSG00000143067	ZNF697	2881	2.60E-18	2.62E-19	0.72	0.07	0.21	2.93
ENSG00000203782	LORICRIN	3572	5.25E-02	4.97E-03	0.74	0.07	0.14	1.96
ENSG00000077254	USP33	4552	4.54E-05	4.72E-05	0.43	0.44	0.13	0.30
ENSG00000116266	STXBP3	4434	1.07E-01	1.24E-02	0.72	0.08	0.08	0.92
ENSG00000121940	CLCC1	4311	7.44E-10	6.81E-11	0.85	0.08	0.07	0.92

nsnps: Number of overlapping SNPs between for each locus between the eqtl and the GWAS traits
PP.<hypothesis>: The posterior probability for each coloc hypothesis
ratio_PPH4_PPH3: The ratio of the H4 and H3 posterior porobabilities (ratio = H4/H3

eTable 8. Candidate variants analysis.

Nearest gene	SNP	MAF	BETA	SE	P-value	Direction	Publication	PMID
ANKK1	rs1800497	0.21	0.24	0.09	8.89E-03	+++--	Rieck et al. 2012	23171335
ANKK1	rs2734849	0.50	0.18	0.08	2.11E-02	+++++	Rieck et al. 2012	23171335
BDNF	rs6265	0.18	0.19	0.10	4.95E-02	+++-+	Foltynie et al. 2009<br>Kusters et al. 2018	18977816<br>29191473
DRD2	rs2283265	0.17	0.16	0.10	1.06E-01	+++--	Rieck et al. 2012	23171335
DRD2	rs6277	0.46	0.08	0.08	2.73E-01	----+	Rieck et al. 2012	23171335
DRD2	rs1076560	0.17	0.15	0.10	1.42E-01	+++--	Rieck et al. 2012	23171335
PRKCA	rs4790904	0.22	-0.14	0.10	1.43E-01	-++++	Martin-Flores et al. 2018	29992529
RPS6KB1	rs1292034	0.42	-0.13	0.08	1.08E-01	-----	Martin-Flores et al. 2018	29992529
OPRM1	rs1799971	0.12	-0.13	0.12	3.04E-01	+-+++	Strong et al. 2006	16435402
EIF4EBP2	rs1043098	0.49	0.06	0.08	4.67E-01	+-+--	Martin-Flores et al. 2018	29992529
SLC6A3	rs393795	0.20	0.07	0.10	4.72E-01	-++++	Kaplan et al. 2014<br>Purcaro et al. 2018	24633632<br>30316985

Nearest gene	SNP	MAF	BETA	SE	P-value	Direction	Publication	PMID
RICTOR	rs2043112	0.40	0.05	0.08	5.50E-01	+++-+	Martin-Flores et al. 2018	29992529
HRAS	rs12628	0.35	-0.04	0.08	5.89E-01	+-++-	Martin-Flores et al. 2018	29992529
RPS6KA2	rs6456121	0.30	0.04	0.08	6.29E-01	++--+	Martin-Flores et al. 2018	29992529
COMT	rs4680	0.47	-0.03	0.08	6.65E-01	++---	Bialecka et al. 2004<br>de Lau et al. 2011<br>Hao et al. 2014<br>Cheshire al. 2014	15355491<br>24008922<br>25034874<br>22083803
PRKN	rs1801582	0.16	-0.04	0.11	7.01E-01	-+-++	Martin-Flores et al. 2018	29992529
FCHSD1	rs456998	0.49	-0.03	0.08	7.17E-01	+-+-+	Martin-Flores et al. 2018	29992529
DRD3	rs6280	0.33	0.02	0.08	7.63E-01	+-+-+	Lee et al. 2011	20945430
ADORA2A	rs3761422	0.37	0.02	0.08	7.71E-01	+-++-	Rieck et al. 2015	25872644
ADORA2A	rs2298383	0.40	0.02	0.08	8.39E-01	-+-++	Rieck et al. 2015	25872644
HOMER1	rs4704559	0.09	-0.03	0.13	8.31E-01	+++--	Schumacher-Schuh et al. 2014	24126708

Direction: Indicates the directionality of the effect of the variant across substudies included on each study.

eTable9. List of DJ-1, and PARKIN intergenic variants with a significance value lower than 0.05 on the raw GWAS meta-analysis

SNP	CHR	BP	A1	A2	AF	HR	Beta	SE	P	N	GENE
rs113276175	6	162600287	a	g	0.9827	0.22723	-1.4818	0.395	0.00018	601	DJ1
rs75744512	6	162326363	a	g	0.0106	4.3991	1.4814	0.4411	0.00078	696	DJ1
rs144098700	6	162069891	a	g	0.9849	0.38612	-0.9516	0.3047	0.00179	1708	DJ1
rs73782962	6	162075343	a	g	0.0159	2.32751	0.8448	0.2723	0.00192	1991	DJ1
rs111329397	6	162171336	a	c	0.0211	3.86013	1.3507	0.462	0.00346	1220	DJ1
rs113585246	6	162171423	a	g	0.0211	3.86013	1.3507	0.462	0.00346	1220	DJ1
rs112904254	6	162172584	a	c	0.0211	3.86013	1.3507	0.462	0.00346	1220	DJ1
rs73782959	6	162062632	t	g	0.0154	2.23468	0.8041	0.2813	0.00426	1991	DJ1
rs12111122	6	162068076	a	t	0.0154	2.23468	0.8041	0.2813	0.00426	1991	DJ1
rs57985302	6	162071287	a	g	0.0156	2.22888	0.8015	0.2813	0.00438	1991	DJ1
rs56403254	6	162071717	a	g	0.0156	2.22888	0.8015	0.2813	0.00438	1991	DJ1
rs143041505	6	162074039	a	c	0.9844	0.44866	-0.8015	0.2813	0.00438	1991	DJ1

SNP	CHR	BP	A1	A2	AF	HR	Beta	SE	P	N	GENE
rs6912219	6	162074100	a	c	0.9844	0.44866	-0.8015	0.2813	0.00438	1991	DJ1
rs189617732	6	162535958	a	g	0.9287	0.48384	-0.726	0.256	0.00457	524	DJ1
rs6908330	6	162943061	a	c	0.8908	0.72044	-0.3279	0.1164	0.00484	2687	DJ1
rs2846480	6	162946970	t	c	0.8908	0.72044	-0.3279	0.1164	0.00484	2687	DJ1
rs2022998	6	162941823	t	c	0.88	0.7287	-0.3165	0.1124	0.00486	2687	DJ1
rs2846482	6	162946152	a	g	0.1099	1.38472	0.3255	0.1164	0.00518	2687	DJ1
rs141726555	6	162250488	t	c	0.02	4.82109	1.573	0.5642	0.0053	241	DJ1
rs6455760	6	162110497	a	g	0.1245	1.3477	0.2984	0.1086	0.00598	2687	DJ1
rs182178663	6	162102507	a	g	0.0121	4.63947	1.5346	0.5599	0.00613	1014	DJ1
rs150977839	6	162076149	t	c	0.9847	0.44731	-0.8045	0.2938	0.00617	1991	DJ1
rs9355901	6	161858094	t	c	0.2626	1.26226	0.2329	0.0853	0.00633	2687	DJ1
rs138643736	6	161825450	t	c	0.0126	44.2564	3.79	1.4047	0.00697	77	DJ1
rs76437736	6	162385389	a	c	0.9833	0.20458	-1.5868	0.5941	0.00756	318	DJ1
rs147654033	6	162051481	t	g	0.0136	2.366	0.8612	0.3232	0.00771	1708	DJ1

SNP	CHR	BP	A1	A2	AF	HR	Beta	SE	P	N	GENE
rs113276175	6	162600287	a	g	0.9827	0.22723	-1.4818	0.395	0.00018	601	PARKN
rs75744512	6	162326363	a	g	0.0106	4.3991	1.4814	0.4411	0.00078	696	PARKN
rs144098700	6	162069891	a	g	0.9849	0.38612	-0.9516	0.3047	0.00179	1708	PARKN
rs73782962	6	162075343	a	g	0.0159	2.32751	0.8448	0.2723	0.00192	1991	PARKN
rs111329397	6	162171336	a	c	0.0211	3.86013	1.3507	0.462	0.00346	1220	PARKN
rs113585246	6	162171423	a	g	0.0211	3.86013	1.3507	0.462	0.00346	1220	PARKN
rs112904254	6	162172584	a	c	0.0211	3.86013	1.3507	0.462	0.00346	1220	PARKN
rs73782959	6	162062632	t	g	0.0154	2.23468	0.8041	0.2813	0.00426	1991	PARKN
rs12111122	6	162068076	a	t	0.0154	2.23468	0.8041	0.2813	0.00426	1991	PARKN
rs57985302	6	162071287	a	g	0.0156	2.22888	0.8015	0.2813	0.00438	1991	PARKN
rs56403254	6	162071717	a	g	0.0156	2.22888	0.8015	0.2813	0.00438	1991	PARKN
rs143041505	6	162074039	a	c	0.9844	0.44866	-0.8015	0.2813	0.00438	1991	PARKN
rs6912219	6	162074100	a	c	0.9844	0.44866	-0.8015	0.2813	0.00438	1991	PARKN
rs189617732	6	162535958	a	g	0.9287	0.48384	-0.726	0.256	0.00457	524	PARKN

SNP	CHR	BP	A1	A2	AF	HR	Beta	SE	P	N	GENE
rs6908330	6	162943061	a	c	0.8908	0.72044	-0.3279	0.1164	0.00484	2687	PARKN
rs2846480	6	162946970	t	c	0.8908	0.72044	-0.3279	0.1164	0.00484	2687	PARKN
rs2022998	6	162941823	t	c	0.88	0.7287	-0.3165	0.1124	0.00486	2687	PARKN
rs2846482	6	162946152	a	g	0.1099	1.38472	0.3255	0.1164	0.00518	2687	PARKN
rs141726555	6	162250488	t	c	0.02	4.82109	1.573	0.5642	0.0053	241	PARKN
rs6455760	6	162110497	a	g	0.1245	1.3477	0.2984	0.1086	0.00598	2687	PARKN
rs182178663	6	162102507	a	g	0.0121	4.63947	1.5346	0.5599	0.00613	1014	PARKN
rs150977839	6	162076149	t	c	0.9847	0.44731	-0.8045	0.2938	0.00617	1991	PARKN
rs9355901	6	161858094	t	c	0.2626	1.26226	0.2329	0.0853	0.00633	2687	PARKN
rs138643736	6	161825450	t	c	0.0126	44.2564	3.79	1.4047	0.00697	77	PARKN
rs76437736	6	162385389	a	c	0.9833	0.20458	-1.5868	0.5941	0.00756	318	PARKN
rs147654033	6	162051481	t	g	0.0136	2.366	0.8612	0.3232	0.00771	1708	PARKN
rs117765565	6	162871441	a	t	0.1003	0.64927	-0.4319	0.1628	0.00797	2445	PARKN
rs1801474	6	162622197	t	c	0.0146	2.19614	0.7867	0.297	0.00808	2687	PARKN

SNP	CHR	BP	A1	A2	AF	HR	Beta	SE	P	N	GENE
rs9458505	6	162725665	t	c	0.0913	1.42333	0.353	0.1339	0.00838	2404	PARKN
rs6914057	6	162871262	t	c	0.9005	1.4981	0.4042	0.1538	0.00857	2687	PARKN
rs12662364	6	161855965	a	g	0.2642	1.24995	0.2231	0.085	0.00867	2687	PARKN
rs62437986	6	162582554	t	c	0.986	0.2644	-1.3303	0.5092	0.00899	1297	PARKN
rs55912218	6	162307775	t	taa	0.7153	1.86526	0.6234	0.2409	0.00966	281	PARKN
rs149634732	6	162047729	t	c	0.0136	2.10244	0.7431	0.2872	0.00968	1991	PARKN
rs9347542	6	162235691	t	c	0.4193	1.46594	0.3825	0.1486	0.01006	524	PARKN
rs151243521	6	162460413	t	c	0.9833	0.2195	-1.5164	0.5893	0.01007	318	PARKN
rs112858840	6	163137317	c	g	0.9833	0.4205	-0.8663	0.342	0.0113	1220	PARKN
rs57374961	6	162615335	c	g	0.0151	2.09552	0.7398	0.2931	0.0116	2687	PARKN
rs78718632	6	162877658	a	g	0.1009	0.68537	-0.3778	0.1514	0.01258	2687	PARKN
rs74475107	6	162877889	a	g	0.8991	1.45907	0.3778	0.1514	0.01258	2687	PARKN
rs143941834	6	162640507	a	g	0.9842	0.29724	-1.2132	0.4873	0.01278	524	PARKN
rs75342128	6	162652000	a	g	0.9842	0.29724	-1.2132	0.4873	0.01278	524	PARKN

SNP	CHR	BP	A1	A2	AF	HR	Beta	SE	P	N	GENE
rs76146820	6	162707685	a	g	0.0158	3.36423	1.2132	0.4873	0.01278	524	PARKN
rs9365407	6	162785031	a	g	0.8226	0.78718	-0.2393	0.0964	0.01305	2687	PARKN
rs6941157	6	162936188	t	c	0.0565	1.44672	0.3693	0.1488	0.01307	2687	PARKN
rs12214138	6	162107046	t	c	0.9638	0.64076	-0.4451	0.1829	0.01494	2687	PARKN
rs118180252	6	162097162	t	c	0.0233	1.65367	0.503	0.2073	0.01524	2687	PARKN
rs60095244	6	162946561	t	c	0.0564	1.42975	0.3575	0.1489	0.01636	2687	PARKN
rs60074916	6	162946564	t	c	0.0564	1.42975	0.3575	0.1489	0.01636	2687	PARKN
rs16892937	6	162125691	t	c	0.0188	1.74072	0.5543	0.2312	0.01651	2687	PARKN
rs192576507	6	162715710	t	c	0.9916	0.02812	-3.5714	1.4967	0.01703	77	PARKN
rs80330859	6	162859951	c	g	0.9031	1.44037	0.3649	0.1533	0.01726	2687	PARKN
rs59996420	6	162122039	a	g	0.9811	0.57782	-0.5485	0.2312	0.01767	2687	PARKN
rs62430696	6	162752612	t	c	0.9808	0.56666	-0.568	0.24	0.01795	2687	PARKN
rs62430697	6	162753224	t	c	0.0192	1.76473	0.568	0.24	0.01795	2687	PARKN
rs9458572	6	162935431	t	c	0.0507	1.44023	0.3648	0.1543	0.0181	2687	PARKN

SNP	CHR	BP	A1	A2	AF	HR	Beta	SE	P	N	GENE
rs374239029	6	162777567	a	at	0.7438	1.87142	0.6267	0.2653	0.01816	283	PARKN
rs73597197	6	162124704	t	c	0.9808	0.58077	-0.5434	0.2313	0.01882	2687	PARKN
rs9365410	6	162795058	t	c	0.061	1.40649	0.3411	0.1462	0.0196	2687	PARKN
rs79565809	6	162119637	a	g	0.0189	1.71412	0.5389	0.2312	0.01974	2687	PARKN
rs1016085	6	162242404	t	c	0.7634	0.81595	-0.2034	0.0873	0.01979	2687	PARKN
rs116357950	6	161970866	t	c	0.0338	1.64001	0.4947	0.2125	0.01992	2687	PARKN
rs186614596	6	162534882	t	c	0.0589	0.25576	-1.3635	0.5862	0.02001	524	PARKN
rs111367465	6	162535731	a	c	0.0589	0.25576	-1.3635	0.5862	0.02001	524	PARKN
rs62437978	6	162573952	a	g	0.0084	29.45312	3.3828	1.4632	0.02078	77	PARKN
rs140706721	6	162477163	t	c	0.0085	3.42123	1.23	0.5357	0.02166	773	PARKN
rs146099223	6	162563818	a	g	0.0085	3.42123	1.23	0.5357	0.02166	773	PARKN
rs150553548	6	162695552	a	t	0.0104	2.66153	0.9789	0.4264	0.02169	1390	PARKN
rs144698234	6	162715035	a	g	0.0104	2.66153	0.9789	0.4264	0.02169	1390	PARKN
rs79026473	6	162097898	a	c	0.9761	0.62744	-0.4661	0.2033	0.02185	2687	PARKN

SNP	CHR	BP	A1	A2	AF	HR	Beta	SE	P	N	GENE
rs9458348	6	162149539	t	c	0.7107	0.82456	-0.1929	0.0841	0.02186	2687	PARKN
rs74922818	6	162696500	a	c	0.0107	2.65727	0.9773	0.4264	0.02191	1390	PARKN
rs138089003	6	162354879	t	g	0.0168	7.86797	2.0628	0.9004	0.02197	77	PARKN
rs138771484	6	162432979	t	c	0.979	0.1271	-2.0628	0.9004	0.02197	77	PARKN
rs60883055	6	162611302	t	c	0.9769	0.57132	-0.5598	0.2451	0.02238	2687	PARKN
rs140432232	6	162843810	c	g	0.9028	1.41397	0.3464	0.1519	0.0226	2687	PARKN
rs192586459	6	162876746	t	c	0.1081	0.45841	-0.78	0.3422	0.02263	524	PARKN
rs188082227	6	162723904	a	t	0.0168	10.47614	2.3491	1.033	0.02296	77	PARKN
rs6930628	6	162110806	a	g	0.0218	1.65004	0.5008	0.2203	0.02301	2687	PARKN
rs16892913	6	162112309	a	t	0.9782	0.60605	-0.5008	0.2203	0.02301	2687	PARKN
rs76290633	6	162116665	a	g	0.0218	1.65004	0.5008	0.2203	0.02301	2687	PARKN
rs4709541	6	162103294	a	g	0.2062	0.78852	-0.2376	0.1046	0.02306	2687	PARKN
rs11751911	6	162235611	t	c	0.2516	0.80961	-0.2112	0.0931	0.02329	2687	PARKN
rs4708931	6	162190804	a	c	0.949	0.65423	-0.4243	0.188	0.02402	1914	PARKN

SNP	CHR	BP	A1	A2	AF	HR	Beta	SE	P	N	GENE
rs4455643	6	161852968	t	c	0.7346	0.82572	-0.1915	0.085	0.02437	2687	PARKN
rs9347509	6	161853203	a	g	0.7346	0.82572	-0.1915	0.085	0.02437	2687	PARKN
rs62436001	6	162391014	t	c	0.0159	2.99936	1.0984	0.4884	0.0245	1220	PARKN
rs77460805	6	162773895	c	g	0.892	1.37479	0.3183	0.1423	0.02535	2687	PARKN
rs10755584	6	162469736	t	c	0.8648	0.77942	-0.2492	0.1115	0.02542	2687	PARKN
rs117230217	6	162296735	t	c	0.0283	2.7871	1.025	0.4627	0.02672	524	PARKN
rs9295181	6	162481460	t	c	0.5783	0.83937	-0.1751	0.0793	0.02729	2687	PARKN
rs568459658	6	162192301	t	c	0.9833	0.54313	-0.6104	0.2772	0.02765	2687	PARKN
rs557347438	6	162227423	a	c	0.0168	22.20017	3.1001	1.412	0.02812	77	PARKN
rs62429614	6	162940960	t	c	0.9816	0.5351	-0.6253	0.2871	0.0294	2610	PARKN
rs78907851	6	162727657	t	c	0.0582	1.38085	0.3227	0.1486	0.02984	2687	PARKN
rs9364650	6	162728256	a	c	0.9418	0.72419	-0.3227	0.1486	0.02984	2687	PARKN
rs75157574	6	162434018	t	c	0.025	1.7291	0.5476	0.2541	0.03112	1991	PARKN
rs79220797	6	162835227	a	g	0.9026	1.38292	0.3242	0.1506	0.03133	2687	PARKN

SNP	CHR	BP	A1	A2	AF	HR	Beta	SE	P	N	GENE
rs10945811	6	162738512	a	g	0.0582	1.37754	0.3203	0.1488	0.03134	2687	PARKN
rs9355898	6	161847010	t	c	0.2694	0.67086	-0.3992	0.1858	0.03164	524	PARKN
rs75923621	6	162830552	a	t	0.097	0.72368	-0.3234	0.1506	0.03173	2687	PARKN
rs79699210	6	162834650	a	g	0.097	0.72368	-0.3234	0.1506	0.03173	2687	PARKN
rs74892485	6	162834786	t	c	0.097	0.72368	-0.3234	0.1506	0.03173	2687	PARKN
rs78174510	6	162839960	t	c	0.9026	1.38182	0.3234	0.1506	0.03175	2687	PARKN
rs12210160	6	161814907	a	g	0.897	1.34299	0.2949	0.1374	0.0318	2687	PARKN
rs2186815	6	162886686	a	g	0.052	1.40214	0.338	0.1575	0.03186	2687	PARKN
rs78964633	6	162837366	t	c	0.0972	0.7239	-0.3231	0.1506	0.03193	2687	PARKN
rs11753929	6	162088198	t	c	0.9785	0.59156	-0.525	0.245	0.03211	2687	PARKN
rs71653628	6	161770811	t	g	0.044	1.44773	0.37	0.1727	0.03219	2687	PARKN
rs146512832	6	162220484	c	g	0.9876	0.25173	-1.3794	0.6462	0.0328	283	PARKN
rs62430699	6	162774531	a	g	0.01	5.76036	1.751	0.8253	0.03387	241	PARKN
rs747295	6	162888355	t	c	0.9061	0.77136	-0.2596	0.1227	0.03445	2687	PARKN

SNP	CHR	BP	A1	A2	AF	HR	Beta	SE	P	N	GENE
rs75888372	6	162804109	t	g	0.9029	1.37383	0.3176	0.1505	0.03485	2687	PARKN
rs114696305	6	162812684	a	g	0.0974	0.72826	-0.3171	0.1504	0.03494	2687	PARKN
rs35029799	6	162283430	a	g	0.9207	0.76208	-0.2717	0.1294	0.03577	2687	PARKN
rs6455759	6	162106439	t	c	0.2119	0.80541	-0.2164	0.1031	0.03581	2687	PARKN
rs542536086	6	163141639	t	c	0.0117	4.93573	1.5965	0.7635	0.03653	241	PARKN
rs78648977	6	162803458	t	g	0.01	5.47504	1.7002	0.8151	0.03699	241	PARKN
rs9347599	6	162611892	t	c	0.0145	1.83639	0.6078	0.2915	0.03707	2687	PARKN
rs9355933	6	162190430	a	t	0.0505	1.48676	0.3966	0.1904	0.03719	1914	PARKN
rs4708930	6	162190759	t	c	0.0505	1.48676	0.3966	0.1904	0.03719	1914	PARKN
rs1001091	6	162237057	a	g	0.7275	1.20889	0.1897	0.091	0.03719	2687	PARKN
rs2849564	6	162459664	t	c	0.3709	1.17998	0.1655	0.0794	0.03728	2687	PARKN
rs79084151	6	162832167	t	g	0.8978	1.35256	0.302	0.1452	0.03758	2687	PARKN
rs1122327	6	162235108	t	c	0.3028	1.1891	0.1732	0.0833	0.0376	2687	PARKN
rs74778695	6	162931570	a	g	0.0229	2.35608	0.857	0.4123	0.03768	601	PARKN

SNP	CHR	BP	A1	A2	AF	HR	Beta	SE	P	N	GENE
rs6914080	6	162871297	t	c	0.6423	0.84764	-0.1653	0.0795	0.03769	2687	PARKN
rs9295150	6	161885096	a	g	0.0213	1.64132	0.4955	0.2386	0.03781	2610	PARKN
rs9458453	6	162502852	a	t	0.5467	1.17774	0.1636	0.0788	0.03794	2687	PARKN
rs200316663	6	162697733	g	gtgtc	0.9466	2.68451	0.9875	0.4759	0.03797	524	PARKN
rs192572757	6	162531271	t	c	0.979	0.09673	-2.3358	1.1288	0.03851	77	PARKN
rs141512658	6	162552268	a	c	0.021	10.33773	2.3358	1.1288	0.03851	77	PARKN
rs4314474	6	162518553	t	c	0.3262	1.39766	0.3348	0.162	0.03869	524	PARKN
rs75856612	6	162513392	a	g	0.9883	0.19311	-1.6445	0.7973	0.03916	241	PARKN
rs73013498	6	162139284	a	g	0.2006	1.21933	0.1983	0.0963	0.0395	2687	PARKN
rs2851401	6	162459762	t	c	0.4967	1.1721	0.1588	0.0772	0.03984	2687	PARKN
rs7758666	6	162249776	a	c	0.2845	1.19017	0.1741	0.0848	0.04005	2687	PARKN
rs12205900	6	162094674	a	g	0.0339	1.4755	0.389	0.1901	0.04071	2687	PARKN
rs60798458	6	161847112	a	c	0.1307	1.7369	0.5521	0.2705	0.04126	283	PARKN
rs9365409	6	162792379	t	c	0.1907	1.20852	0.1894	0.0931	0.04194	2687	PARKN

SNP	CHR	BP	A1	A2	AF	HR	Beta	SE	P	N	GENE
rs117076984	6	162991852	c	g	0.0302	1.56721	0.4493	0.2215	0.04248	2687	PARKN
rs112765480	6	162770514	c	g	0.9805	0.60854	-0.4967	0.2459	0.04342	2687	PARKN
rs12193568	6	162557719	a	g	0.7507	0.83644	-0.1786	0.0886	0.04393	2687	PARKN
rs4709569	6	162503148	a	c	0.5455	1.17128	0.1581	0.0787	0.04455	2687	PARKN
rs1001090	6	162237100	a	g	0.7292	1.19973	0.1821	0.0909	0.04516	2687	PARKN
rs9458452	6	162501019	t	c	0.5448	1.17	0.157	0.0788	0.04621	2687	PARKN
rs13217906	6	161804399	a	g	0.1024	0.76231	-0.2714	0.1363	0.04647	2687	PARKN
rs10806755	6	162495315	a	g	0.2234	1.41468	0.3469	0.1745	0.04677	524	PARKN
rs78600213	6	161939935	c	g	0.0161	1.74334	0.5558	0.2796	0.04686	2687	PARKN
rs1893545	6	162451368	t	g	0.2005	1.21046	0.191	0.0966	0.04804	2687	PARKN
rs79948313	6	162725598	a	t	0.0592	1.33442	0.2885	0.1462	0.04845	2687	PARKN
rs138485300	6	162463415	a	c	0.0097	2.19416	0.7858	0.3983	0.0485	2163	PARKN
rs12213394	6	161797681	a	g	0.1021	0.76414	-0.269	0.1364	0.04859	2687	PARKN
rs12213397	6	161797690	t	g	0.1021	0.76414	-0.269	0.1364	0.04859	2687	PARKN

SNP	CHR	BP	A1	A2	AF	HR	Beta	SE	P	N	GENE
rs10806754	6	162495290	t	g	0.7747	0.70957	-0.3431	0.1741	0.04877	524	PARKN
rs117406278	6	162431177	t	c	0.9295	0.64025	-0.4459	0.2267	0.04914	524	PARKN
rs13191078	6	162476705	a	c	0.4528	0.85607	-0.1554	0.079	0.04927	2687	PARKN
rs1018462	6	162141896	a	g	0.6623	0.85266	-0.1594	0.0811	0.04945	2687	PARKN
rs146701725	6	162725858	c	g	0.0142	3.35483	1.2104	0.6161	0.04945	282	PARKN
rs2849565	6	162452162	a	g	0.1475	1.23937	0.2146	0.1093	0.04947	2687	PARKN
rs139103004	6	162416907	t	c	0.0138	2.18475	0.7815	0.3978	0.04949	1014	PARKN
rs9458252	6	161860169	t	c	0.5577	0.8559	-0.1556	0.0795	0.05029	2687	PARKN

eTable 10. Table of SNPs used to derive the GRS in the TPD cohort

SNP	CHR	BP	MAF	BETA	SE	P-value
4:32435284	4	32435284	0.0194	1.1245	0.1866	1.673e-09
16:17044975	16	17044975	0.9764	-1.14	0.1962	6.265e-09
1:53778300	1	53778300	0.0233	1.0163	0.1796	1.527e-08
1:168645690	1	168645690	0.0466	0.7671	0.1423	7.037e-08
1:80950480	1	80950480	0.9548	-0.7594	0.1419	8.692e-08
9:22664277	9	22664277	0.0143	1.2588	0.2356	9.192e-08
2:47299166	2	47299166	0.019	1.0791	0.205	1.416e-07
13:73750127	13	73750127	0.0672	0.6937	0.1343	2.41e-07
3:8537619	3	8537619	0.9334	-0.6374	0.1256	3.856e-07
3:4714509	3	4714509	0.0287	0.8517	0.1693	4.901e-07
2:242660630	2	242660630	0.0144	1.237	0.2459	4.905e-07
12:48124937	12	48124937	0.0187	1.0675	0.2124	4.989e-07
5:108270767	5	108270767	0.0137	1.2506	0.2509	6.221e-07

SNP	CHR	BP	MAF	BETA	SE	P-value
8:138767782	8	138767782	0.0348	0.8365	0.168	6.423e-07
14:22023873	14	22023873	0.9868	-1.1512	0.2321	7.04e-07
7:46390116	7	46390116	0.0224	0.9524	0.1936	8.679e-07
20:51996645	20	51996645	0.0139	1.0939	0.2227	9.062e-07
2:176515033	2	176515033	0.0596	0.6755	0.1376	9.191e-07
4:41614236	4	41614236	0.9878	-1.2227	0.2496	9.637e-07
7:125755287	7	125755287	0.0321	0.881	0.1801	9.986e-07
11:93486449	11	93486449	0.9842	-1.2365	0.2533	1.055e-06
19:30990206	19	30990206	0.9762	-1.0323	0.2119	1.104e-06
6:16636328	6	16636328	0.0405	0.7637	0.157	1.152e-06
3:124888091	3	124888091	0.9463	-0.6963	0.1432	1.166e-06
1:81453727	1	81453727	0.9875	-1.1372	0.2342	1.204e-06
19:954677	19	954677	0.0266	0.9056	0.1867	1.233e-06
16:78198192	16	78198192	0.2731	0.4042	0.0835	1.287e-06

SNP	CHR	BP	MAF	BETA	SE	P-value
4:64494944	4	64494944	0.0206	0.9275	0.1923	1.414e-06
5:132249144	5	132249144	0.016	1.0617	0.2204	1.462e-06
2:62427404	2	62427404	0.0191	0.9786	0.2032	1.463e-06
15:61097179	15	61097179	0.0174	1.4453	0.3005	1.514e-06
8:103719246	8	103719246	0.0113	1.3437	0.2804	1.656e-06
3:128349376	3	128349376	0.9599	-0.7262	0.1516	1.672e-06
1:106719668	1	106719668	0.0302	0.8321	0.1739	1.707e-06
7:157406470	7	157406470	0.9203	-0.5601	0.1171	1.733e-06
9:124098846	9	124098846	0.0199	0.9737	0.2047	1.972e-06
18:1941742	18	1941742	0.0169	1.039	0.2187	2.035e-06
5:75719143	5	75719143	0.0156	1.2049	0.2538	2.052e-06
13:29849305	13	29849305	0.023	0.8936	0.1883	2.084e-06
13:95085417	13	95085417	0.1269	0.4899	0.1035	2.204e-06
4:94768877	4	94768877	0.0118	1.1815	0.2501	2.317e-06

SNP	CHR	BP	MAF	BETA	SE	P-value
18:54853305	18	54853305	0.023	0.9298	0.1977	2.57e-06
6:115767948	6	115767948	0.0256	0.8527	0.1819	2.765e-06
6:118528190	6	118528190	0.0334	0.8212	0.1756	2.932e-06
13:110061713	13	110061713	0.1381	0.4664	0.0998	2.999e-06
16:686398	16	686398	0.9824	-1.0484	0.2247	3.081e-06
12:131890366	12	131890366	0.0111	1.2191	0.2621	3.301e-06
5:134774168	5	134774168	0.0111	1.4659	0.3159	3.49e-06
4:31553640	4	31553640	0.9863	-1.1423	0.2463	3.531e-06
3:5287334	3	5287334	0.9855	-1.1169	0.2411	3.61e-06
7:157515114	7	157515114	0.0131	1.0623	0.2294	3.629e-06
18:42296688	18	42296688	0.0127	1.216	0.2627	3.662e-06
2:37411014	2	37411014	0.9597	-0.715	0.1544	3.662e-06
3:73128332	3	73128332	0.9876	-1.2229	0.2643	3.728e-06
11:4852271	11	4852271	0.0892	0.5495	0.119	3.902e-06

SNP	CHR	BP	MAF	BETA	SE	P-value
11:99250689	11	99250689	0.0193	0.9499	0.2058	3.906e-06
13:95774697	13	95774697	0.9329	-0.5648	0.1224	3.952e-06
1:78130243	1	78130243	0.2654	0.3958	0.0859	4.034e-06
1:71964730	1	71964730	0.9825	-0.9834	0.2135	4.113e-06
16:20409582	16	20409582	0.9864	-1.1181	0.2432	4.268e-06
9:81601411	9	81601411	0.0207	1.047	0.2278	4.291e-06
11:6636154	11	6636154	0.0133	1.2502	0.2722	4.364e-06
20:6363480	20	6363480	0.3078	0.3717	0.081	4.494e-06
14:59208732	14	59208732	0.1591	0.454	0.099	4.529e-06
8:135945999	8	135945999	0.9825	-1.037	0.2266	4.732e-06
3:82436301	3	82436301	0.0108	1.3701	0.2997	4.853e-06
1:55014822	1	55014822	0.9634	-0.7579	0.1659	4.945e-06

eTable 11. MoCa and UPDRS score comparison between PD-LiD and PD groups

Variable	method	p.value	statistic	PD group mean(sd)	PD-LID group mean(sd)
moca_bl	Wilcoxon rank sum test with continuity correction	2.1e-05	73754.5	25.16(3.31)	26.09(3.56)
moca_visit	Wilcoxon rank sum test with continuity correction	0.01	77249.5	24.34(4.77)	25.35(4.11)
updrs_III_bl	Welch Two Sample t-test	0.15	-1.45	22.2(11.6)	23.72(12.1)
updrs_III_visit	Welch Two Sample t-test	0.25	1.15	31.91(16.7)	30.42(14.0)

moca_bl = Moca average scores for the LiD and PD group at baseline
moca_visit = Moca average scores for the LiD and PD group at the time LiD was developed or at the last visit available
updrsIII_bl = MDS-UPDRS III averages scores for the LiD and PD group at baseline
Updrs_iii_visit = MDS-UPDRS III average scores for the LiD and PD group at the time LiD was developed or at the last visit available

2. eFigures.

eFigure 1. Quality control flowchart

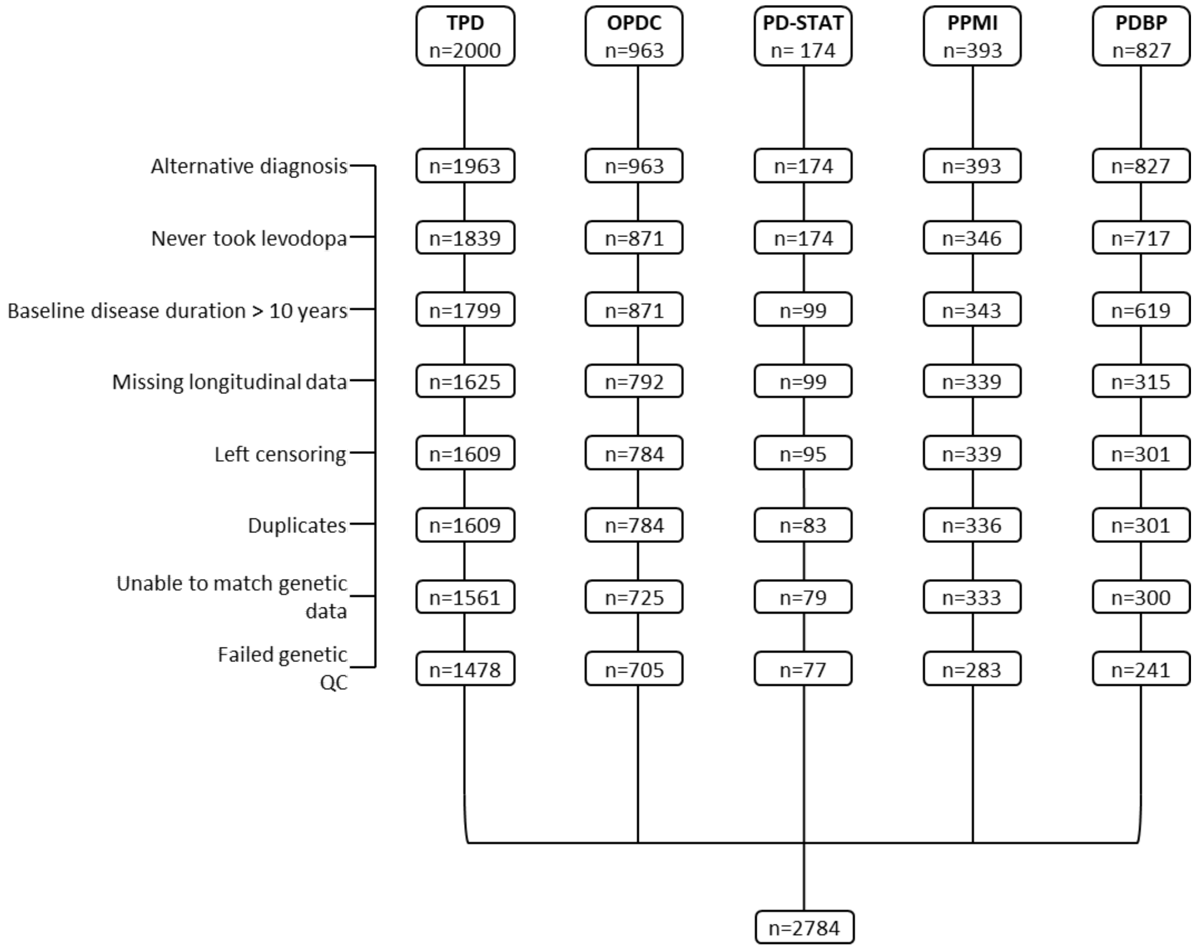

eFigure 2. LiD risk factors Kaplan Meyer curves. Kaplan-Meier curve for Survival probability (LID free probability) based on sex (A), age at onset (AAO) (B), smoking status (C), and smoking status baseline body mass index (BMI). The P-value (P) showing the significance of differences on the survival probability is given on each plot. Number at risk represents the number of PD patients remaining on the study at the different time points (0, 5, 10, 15 years). The colour expansion on each curve represents the confidence interval (CI).

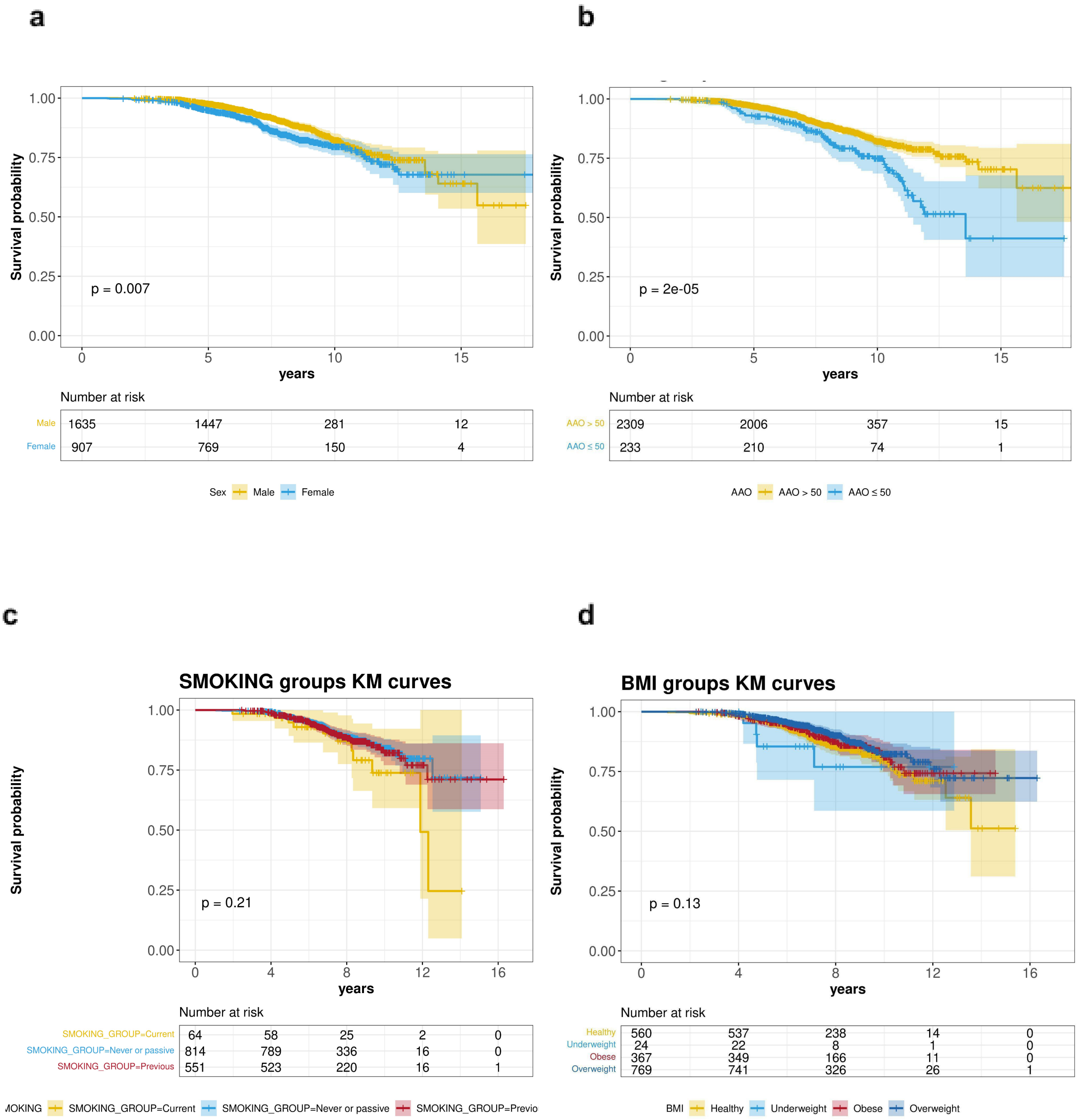

Previous = Previous smoker from baseline
Current = Current smoker at baseline
Never or passive = Never or passive smoker at baseline

eFigure 3. **Power calculation and simulation**. Power calculation and simulation to detect genetic association with time to develop LID as a function of sample size, relative allele frequency (AF), and genetic hazard ratio (GHR). a) Power calculation (y-axis) for the current sample size based on different AFs (x-axis) (graph label) ; b) Power simulation to explore the increase in power (y-axis) to detect lower GHR (graph grid) and relative AFs (graph label) as we increase the sample size (x-axis)

a

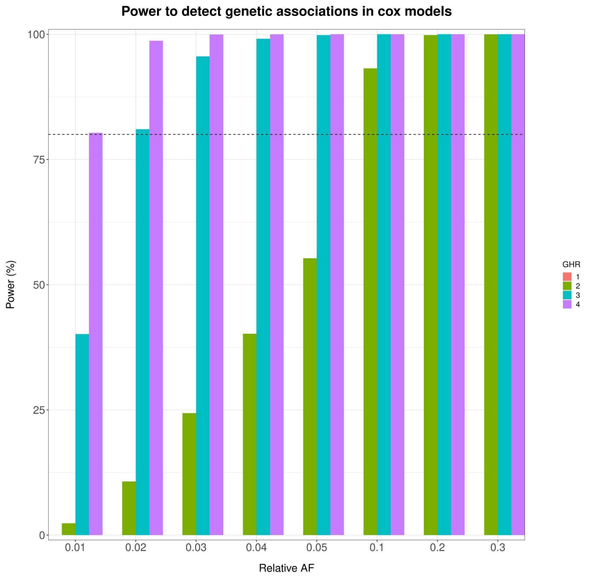

b

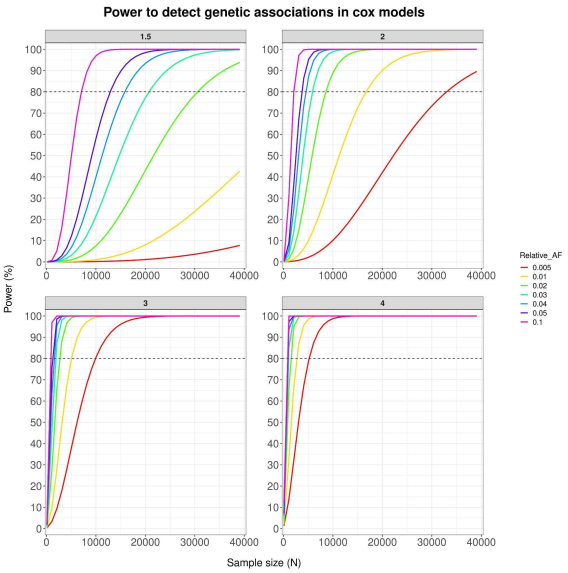

eFigure 4. SNP metrics correlation between the CPH basic and best models. Correlation between SNP regression coefficients (A) and SNP test-statistic (B) in from the basic and the adjusted model. The Pearson correlation coefficient ® and the significance (p) is given.

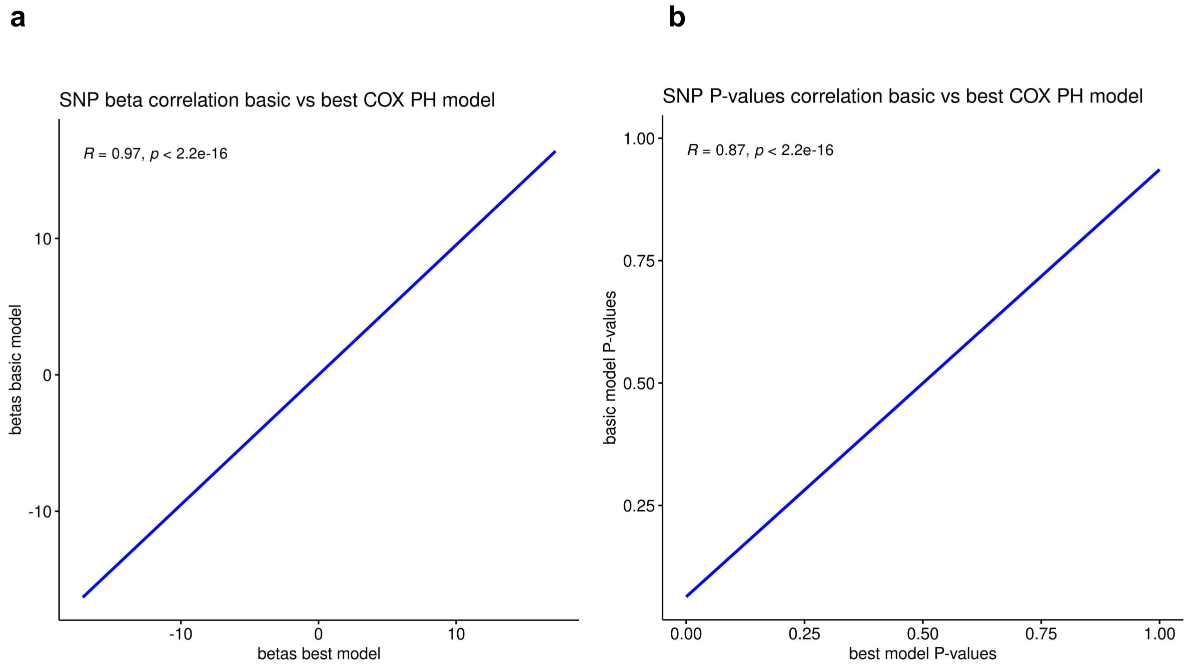

eFigure 5. LRP8 locus fine-mapping and top 5 TFBS marks. From top to bottom, *LRP8* transcript plot, locus plot, the fine-mapping nominated variants across fine-mapping tools, and the top 4 ENCODE TFBS peaks in the *XYLT1* locus. In the locus plot, the SNPs are coloured in red as LD (given by R2) increases, and blue as the LD decreases. In the fine-mapping track, we highlight the SNPs with the highest posterior probabilities for each fine-mapping tool (ABF, FINEMAP, SUSIE, POLYFUN_SUSIE). In addition, we highlight in yellow the Consensus SNP with the highest mean Posterior Probability (mean). The next four rows show the transcription factor binding sites (TFBS) densities (y-axis) measured on different cell lines and laboratories. XGR finds the top 5 transcription factors (TF) with the highest binding activity in the track genomic window. These top 5 TF are displayed in the Assay label.

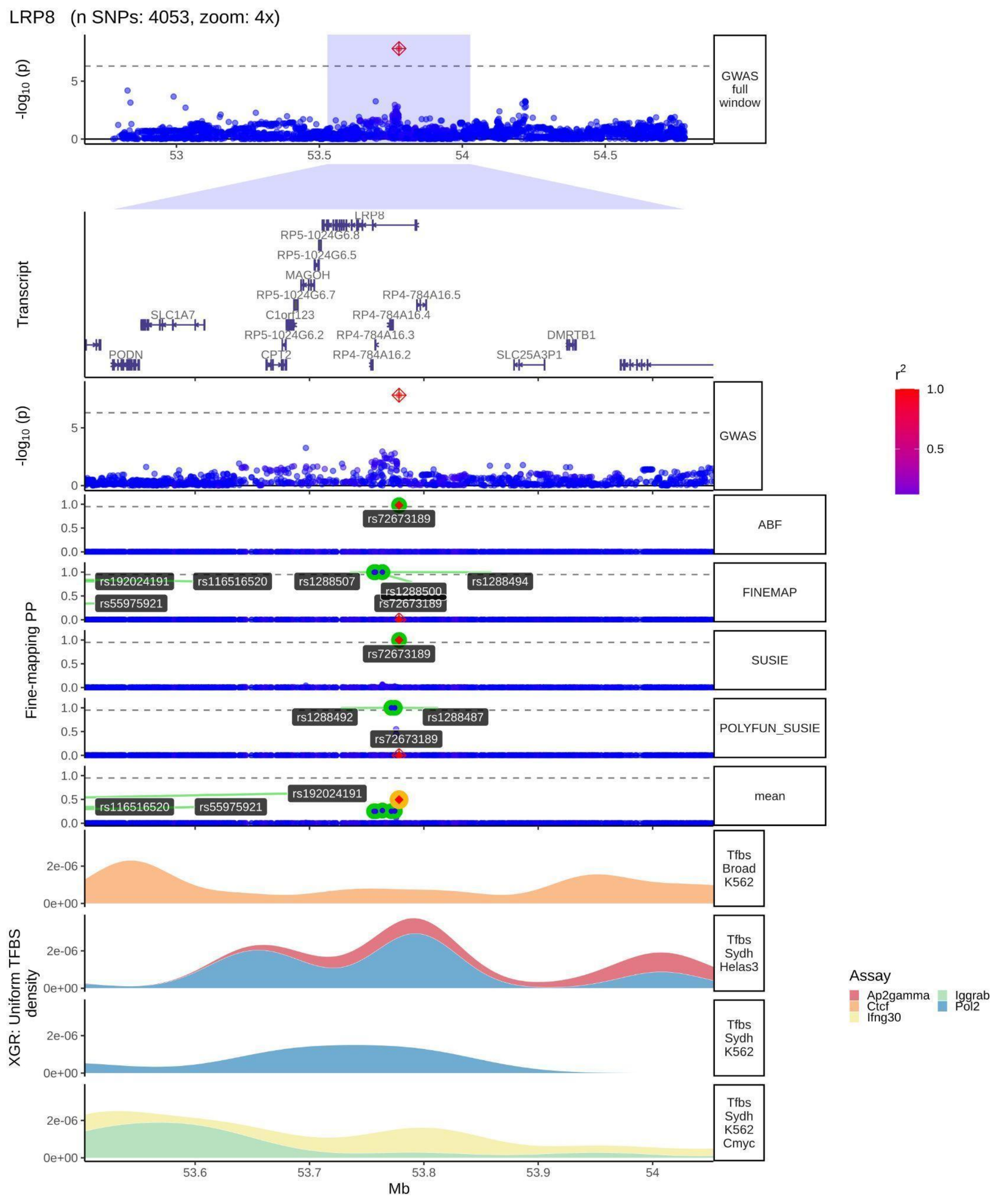

eFigure 6. XYLT1 locus fine-mapping and brain cell type specific regulatory marks. From top to bottom, transcript plot, locus plot, the fine-mapping nominated variants across fine-mapping tools, brain cell type specific regulatory element marks. In the locus plot, the SNPs are coloured in red as LD (given by R2) increases, and blue as the LD decreases. In the fine-mapping track, we highlight the SNPs with the highest posterior probabilities for each fine-mapping tool (ABF, FINEMAP, SUSIE, POLYFUN_SUSIE). In addition, we highlight in yellow the Consensus SNP with the highest mean Posterior Probability (mean). In the cell type specific regulatory element marks, the first 4 rows are the density marks (y-axis) from ATAC-seq assay (in pink), and CHIP-seq assays (H3K27ac in blue, and H3K4me3 in cyan), in astrocytes, microglia, neurons, and oligodendrocytes. The next four rows are the distal anchored chromatin loops (black curves). We see how, only in neurons, there is a chromatin loop forming from the *XYL71* GWS and the fine-mapped consensus variant towards the LRP8 promoter (purple).

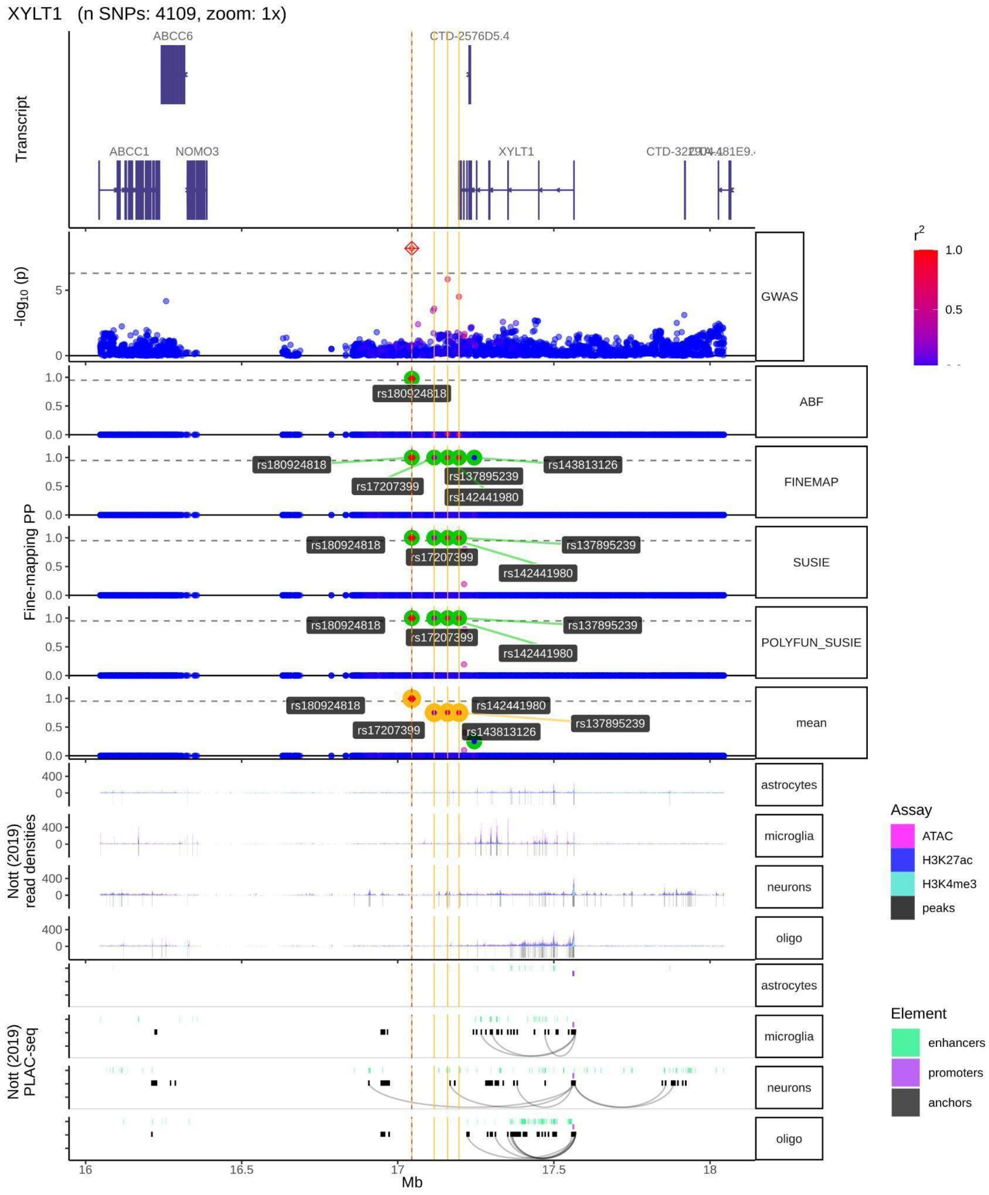

eFigure 7. XYLT1 locus fine-mapping and top 5 TFBS marks. From top to bottom, *XYLT1* transcript plot, locus plot, the fine-mapping nominated variants across fine-mapping tools, and the top 4 ENCODE TFBS peaks in the *XYLT1* locus. In the locus plot, the SNPs are coloured in red as LD (given by R2) increases, and blue as the LD decreases. In the fine-mapping track, we highlight the SNPs with the highest posterior probabilities for each fine-mapping tool (ABF, FINEMAP, SUSIE, POLYFUN_SUSIE). In addition, we highlight in yellow the Consensus SNP with the highest mean Posterior Probability (mean). The next four rows show the transcription factor binding sites (TFBS) densities (y-axis) measured on different cell lines and laboratories. XGR finds the top 5 transcription factors (TF) with the highest binding activity in the track genomic window. These top 5 TF are displayed in the Assay label.

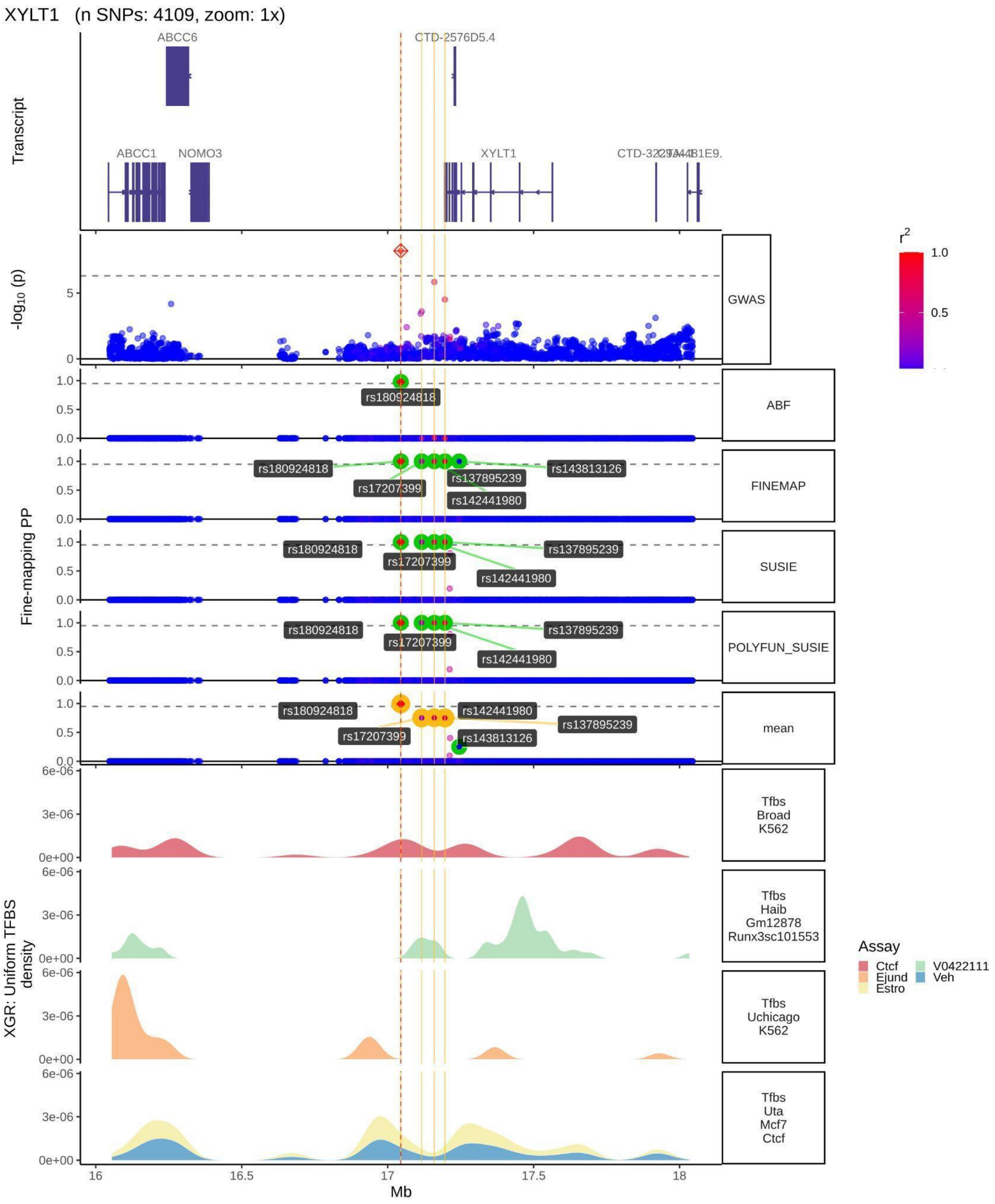

eFigure 8. PRS Receiver operating characteristic (ROC) curves. ROC curves for patient specific PRS measures derived on TPD (A) and OPDC (B) cohorts. The red curve represents the area under the curve (AUC) true positive (sensitivity) versus false positive rate (1 - Specificity) rate.

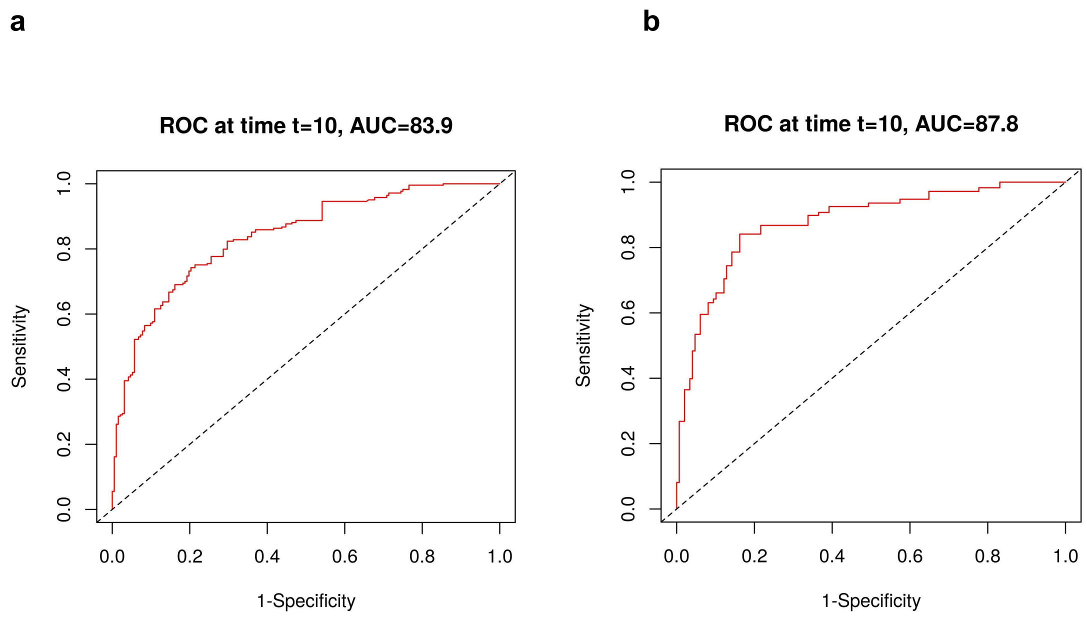

eFigure 9. Precision-recall curves (PROC) and the area under the curve for unweighted data. PROC curves for PD and LiD patients PRS. a) PROC PRS in Tracking Parkinson's and b) PROC PRS in OPDC. The color scale on the right side of the plot gives an indication, which classification threshold results in a certain point on the curve. PR = Precision recall. Precision = Positive predictive value. Recall = Sensitivity

a

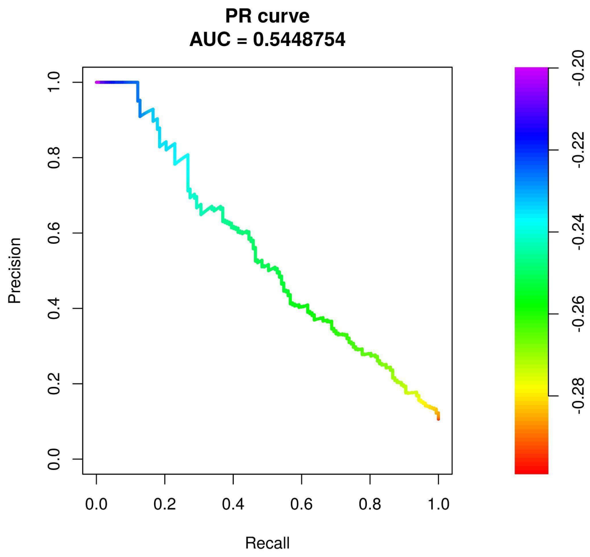

b

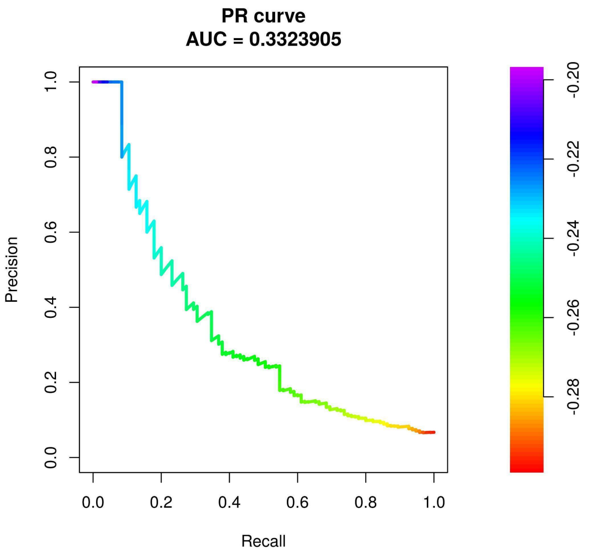

3. eData.

eData 1. List of all covariates included in the stepwise regression model

SEX,MDSUPDRSIII,PC1,PC2,PC3,PC4,PC5,AAO.std,Ldopa_previous,disease_duration_diag,Ldopa_bin,LEDD_levo,LEDD_total_V1,Amantadine_bin_V1,Anticholinergic_bin_V1,COMTI_bin_V1,DA_bin_V1,MAOBI_bin_V1,drug_naive_V1,Centre,dob,disease_duration,disease_duration_diag_cat,time_onset_to_diag,ethnicity_all,demo_QRISK,handedness,religion,marital_status,car_ownership,housing_tenure,years_education_bin,BMI,weight,height,Pulse_rate_lying,Pulse_rate_standing,Systolic_BP_lying,Systolic_BP_standing,Diastolic_BP_lying,Diastolic_BP_standing,SBP_postural_drop,DBP_postural_drop,pulse_postural_drop,orthostatic_hypotension,probability_pd,PDQ8_total,ESS_total,RBD_total,restless_legs,EQ5D_index,EQ5D_vas_score,constip_cat,gastro_total,Leeds_anx_total,Leeds_anx_bin,Leeds_dep_total,Leeds_dep_bin,QUIP_all,MOCA_total,seman_flu_score,scopa_aut_gastro,scopa_aut_urinary,scopa_aut_cardio,scopa_aut_thermo,scopa_aut_puppillo,NMSS_cardio,NMSS_sleep,NMSS_mood,NMSS_perception,NMSS_attention,NMSS_gastro,NMSS_urinary,NMSS_sexual,NMSS_misc,PDSS_total,LEDD_total,drug_naive,Amantadine_bin,Anticholinergic_bin,COMTI_bin,DA_bin,MAOBI_bin,Amantadine_previous,Anticholinergic_previous,COMTI_previous,DA_previous,MAOBI_previous,hoehn_and_yahr_stage,Schwab_england,UPDRS_I_total,UPDRS_II_total,patients_clinical_state,UPDRS_hallucinations,UPDRS_fatigue,UPDRS_pain,UPDRS_constipation,UPDRS_rigidity,UPDRS_bradykinesia,UPDRS_laterality,UPDRS_laterality_bin,side_affected_onset,FSAD_a1,FSAD_a2,FSAD_a3,FSAD_a4,FSAD_a41,FSAD_a42,FSAD_a43,FSAD_a45,FSAD_other_text,SCF_tremor_a11,SCF_tremor_a12,SCF_tremor_a13,SCF_tremor_a14,SCF_tremor_other_text,SCF_rigid_a21,SCF_rigid_a22,SCF_rigid_a23,SCF_rigid_a24,SCF_rigid_a25,SCF_rigid_a26,SCF_rigid_other_text,SCF_brady_a31,SCF_brady_a32,SCF_brady_a33,SCF_brady_a34,SCF_brady_a35,SCF_brady_other_text,SCF_posture_a41,SCF_posture_a42,SCF_posture_a43,SCF_posture_a45,SCF_posture_other_text,SCF_hyperkinesias_a51,SCF_hyperkinesias_a52,SCF_hyperkinesias_a53,SCF_hyperkinesias_a54,SCF_hyperkinesias_other_text,SCF_hemiatrophy_a6,SCF_autonomic_a71,SCF_autonomic_a73,SCF_oculomotor_a8,SCF_eyelid_a9,SCF_otherneuro_Hyperreflexia,SCF_otherneuro_Babinski_sign,SCF_otherneuro_Sensory_deficit,SCF_otherneuro_Amyotrophy,SCF_otherneuro_Limb_apraxia,SCF_otherneuro_Sleep_apnea,SCF_otherneuro_Dysmetria,SCF_otherneuro_other,SCF_response_a11,SCF_tachyphemia_a12,SCF_unusual_Presentation,SCF_unusual_Symptoms,SCF_unusual_Course,SCF_unusual_response,SCF_unusual_Other,SCF_specify_unusual,history_angina,history_heart_fail,history_stroke,history_heart_att,history_diabetes,type_1_diabetes,type_2_diabetes,history_cholesterol,history_high_bp,vascular_cat,heart_disease,vascular_risk,history_bowel,history_prostate,history_breast,history_asthma,history_bronch,history_emphysema,history_arthritis,history_other,adopted,mother_pd,father_pd,matgrand_pd,patgrand_pd,matauun_pd,patauun_pd,sum_fmhx_pd,first_degree_rel_pd,second_degree_rel_pd,recessive_PD_history,dominant_PD_history,mother_stroke,mother_dement_and_alz,father_stroke,father_dement_and_alz,brother_stroke,brother_dement_and_alz,sister_stroke,sister_dement_and_alz,halfsib_dement_and_alz,matgrandfather_stroke,matgrandfather_dement_and_alz,matgrandmother_stroke,matgrandmother_dement_and_alz,patgrandfather_stroke,patgrandfather_dement_and_alz,patgrandmother_stroke,patgrandmother_dement_and_alz,matau_stroke,matau_dement_and_alz,patau_stroke,patau_dement_and_alz,mother_only_stroke,maternal_stroke,father_only_stroke,paternal_stroke,mother_only_dement_and_alz,maternal_dement_and_alz,father_only_dement_and_alz,paternal_dement_and_alz,drugs_cardio,drugs_cardio_group_1,drugs_cardio_group_2,drugs_cardio_group_3,drugs_cardio_group_4,drugs_cardio_group_5,drugs_cardio_group_6,drugs_cardio_group_7,drugs_cardio_group_8,drugs_cardio_group_9,drugs_lipid,drugs_diabetic,drugs_diabetic_insulin,drugs_diabetic_oral,drugs_painkill,drugs_painkill_nsai,drugs_antiplate,drugs_anticoags,drugs_asthmacopd,drugs_asthmacopd_broncho,drugs_asthmacopd_corticost,drugs_laxatives,drugs_bladder,drugs_antidepress,CISIPD_Disability,CISIPD_Motor_complications,CISIPD_Cognitive_status,merqp_smoking,smoke_QRISK,coffee_prior_diag,coffee_current,merqp_a1_work_pesticide,merqp_a2_home_pesticide,merqp_a3_work_solvents,merqp_a4_work_heavymetals,merqp_a5_work_chemicals_fumes,merqp_a5_1,merqp_a12_oophorectomy,merqp_a13_depression,merqp_a14_anxiety,BFI_extra_total,BFI_agree_total,BFI_consci_total,BFI_neuro_total,BFI_open_total,CT_Atrophy,CT_Small_vessel_disease,CT_Lacunar_infarction,CT_Territory_infarction,CT_Hydrocephalus,CT_Other_abnormalit

y,MRI_Atrophy,MRI_Small_vessel_disease,MRI_Lacunar_infarction,MRI_Territory_infarction,MRI_Hydrocephalus,MRI_Other_abnormality,FPCIT_SPECT_res,F_DOPA_PET_res,EQ5D_1,EQ5D_2,EQ5D_3,EQ5D_4,EQ5D_5,LADS_1,LADS_2,LADS_3,LADS_4,LADS_5,LADS_6,LADS_7,LADS_8,LADS_9,LADS_10,LADS_11,LADS_12,constip1_bowelfreq,constip2_laxative,constip3_fruitvegeuse,constip4_exercise,GCSI_a1,GCSI_a2,GCSI_a3,GCSI_a4,GCSI_a5,GCSI_a6,GCSI_a7,GCSI_a8,saut_a1_,saut_a2_,saut_a3_,saut_a4_,saut_a5,saut_a6_,saut_a7_,saut_a14,saut_a15,saut_a16,saut_a17,saut_a18,saut_a19,saut_a20,saut_a21,saut_a8_,saut_a9_,saut_a10,saut_a11,saut_a12,saut_a13,NMSS_severity_score_1,NMSS_severity_score_2,NMSS_severity_score_3,NMSS_severity_score_4,NMSS_severity_score_5,NMSS_severity_score_6,NMSS_severity_score_7,NMSS_severity_score_8,NMSS_severity_score_9,NMSS_severity_score_10,NMSS_severity_score_11,NMSS_severity_score_12,NMSS_severity_score_13,NMSS_severity_score_14,NMSS_severity_score_15,NMSS_severity_score_16,NMSS_severity_score_17,NMSS_severity_score_18,NMSS_severity_score_19,NMSS_severity_score_20,NMSS_severity_score_21,NMSS_severity_score_22,NMSS_severity_score_23,NMSS_severity_score_24,NMSS_severity_score_25,NMSS_severity_score_26,NMSS_severity_score_27,NMSS_severity_score_28,NMSS_severity_score_29,NMSS_severity_score_30,NMSS_frequency_score_1,NMSS_frequency_score_2,NMSS_frequency_score_3,NMSS_frequency_score_4,NMSS_frequency_score_5,NMSS_frequency_score_6,NMSS_frequency_score_7,NMSS_frequency_score_8,NMSS_frequency_score_9,NMSS_frequency_score_10,NMSS_frequency_score_11,NMSS_frequency_score_12,NMSS_frequency_score_13,NMSS_frequency_score_14,NMSS_frequency_score_15,NMSS_frequency_score_16,NMSS_frequency_score_17,NMSS_frequency_score_18,NMSS_frequency_score_19,NMSS_frequency_score_20,NMSS_frequency_score_21,NMSS_frequency_score_22,NMSS_frequency_score_23,NMSS_frequency_score_24,NMSS_frequency_score_25,NMSS_frequency_score_26,NMSS_frequency_score_27,NMSS_frequency_score_28,NMSS_frequency_score_29,NMSS_frequency_score_30,NMSS_1,NMSS_2,NMSS_3,NMSS_4,NMSS_5,NMSS_6,NMSS_7,NMSS_8,NMSS_9,NMSS_10,NMSS_11,NMSS_12,NMSS_13,NMSS_14,NMSS_15,NMSS_16,NMSS_17,NMSS_18,NMSS_19,NMSS_20,NMSS_21,NMSS_22,NMSS_23,NMSS_24,NMSS_25,NMSS_26,NMSS_27,NMSS_28,NMSS_29,NMSS_30,score_BFI_a1_,score_BFI_a6_,score_BFI_a11,score_BFI_a16,score_BFI_a21,score_BFI_a26,score_BFI_a31,score_BFI_a36,score_BFI_a2_,score_BFI_a7_,score_BFI_a12,score_BFI_a17,score_BFI_a22,score_BFI_a27,score_BFI_a32,score_BFI_a37,score_BFI_a42,score_BFI_a3_,score_BFI_a8_,score_BFI_a13,score_BFI_a18,score_BFI_a23,score_BFI_a28,score_BFI_a33,score_BFI_a38,score_BFI_a43,score_BFI_a4_,score_BFI_a9_,score_BFI_a14,score_BFI_a19,score_BFI_a24,score_BFI_a29,score_BFI_a34,score_BFI_a39,score_BFI_a5_,score_BFI_a10,score_BFI_a15,score_BFI_a20,score_BFI_a25,score_BFI_a30,score_BFI_a35,score_BFI_a40,score_BFI_a41,score_BFI_a44,Parents_related,Parents_relationship,Parents_other_relat_state,Person_related,Which_relatives,unusual_present,unusual_present_all,SCF_unusual_Presentation_all,SCF_unusual_Symptoms_all,SCF_unusual_Signs_all,SCF_unusual_Course_all,SCF_unusual_response_all,SCF_unusual_Other_all,prs_nominal.
